# Retrospective analysis reveals significant association of hypoglycemia with tramadol and methadone in contrast to other opioids

**DOI:** 10.1038/s41598-019-48955-y

**Published:** 2019-08-28

**Authors:** Tigran Makunts, Andrew U, Rabia S. Atayee, Ruben Abagyan

**Affiliations:** 1 Skaggs School of Pharmacy and Pharmaceutical Sciences, University of California San Diego, La Jolla, CA USA; 2 UC San Diego Health, Department of Pharmacy, San Diego, USA

**Keywords:** Adverse effects, Pain management, Databases

## Abstract

Tramadol is one of the most commonly used analgesics worldwide, classified as having a low abuse potential by U.S. Drug Enforcement Agency, and often recommended in pain management guidelines. Its pain-relieving mechanism of action is attributed to mild μ-opioid receptor agonism, serotonin and norepinephrine mediated nociception modulation, and N-methyl-D-aspartate receptor, NMDAR, antagonism. However, recent case reports and case-control studies have shown an association between tramadol use and hypoglycemia. The growing concern over increasing tramadol use and unexpected side effects warranted a further comparative and quantitative analysis of tramadol adverse reactions. In this study we analyzed over twelve million reports from United States Food and Drug Administration Adverse Event Reporting System and provided evidence of increased propensity for hypoglycemia in patients taking tramadol when compared to patients taking other opioids, serotonin-norepinephrine reuptake inhibitors, and drugs affecting NMDAR activity. Additionally, we identified that only methadone from the opioid cohort behaves similarly to tramadol and has an association with hypoglycemia.

## Introduction

Tramadol, a synthetic centrally acting weak opioid analgesic approved in 1995, has gradually gained increased worldwide use for acute and chronic pain management due to its low risk of respiratory depression, compared to other opioids^1,2^. Tramadol currently ranks in the top five prescribed opioids and in the top sixty prescribed medications in the United States^3^. According to the 2017 CDC Census Report, prescriptions for tramadol and other synthetic opioids have increased by 88% from 2008 to 2013^4^. Tramadol adverse reaction-related hospital visits have increased two fold since 2005, with female patients accounting for the majority of cases^5,6^. In response to increased tramadol use and its potential for abuse, the Drug Enforcement Agency (DEA) recognized a higher potential of abuse and recategorized tramadol from Schedule V to Schedule IV of the Controlled Substance Act in 2014.

Tramadol’s analgesic effect originates from two distinct mechanisms. It increases the pain threshold by acting on serotonergic and noradrenergic nociception via serotonin and norepinephrine reuptake inhibition (SNRI), and its metabolite, O-desmethyltramadol, acts as a μ-opioid receptor agonist (MOR)^7–9^. Additionally tramadol has an inhibitory effect on N-methyl-D-aspartate receptors (NMDARs)^10^, which are involved in somatic and visceral nociception^11^. Recognized adverse drug reactions (ADRs) of tramadol, common to all opioids, include dizziness/vertigo, nausea, constipation, headache, somnolence, vomiting, pruritus, and others^12^. Rare but serious side effects include serotonin syndrome and increased seizure risk^12^. In addition, recent studies have reported new and unexpected side effect associated with tramadol use.

There have been several case reports describing hypoglycemia induced by tramadol and resolved upon its discontinuation^13–16^.These incidences occurred in both patients with and without diabetes. Hypoglycemia ADR is of great concern since it can lead to many serious complications including neurocognitive dysfunction, retinal cell damage and vision loss, risk of falls, and other complications affecting health and quality of life^17^. In a nested case-control study, Fournier *et al*. identified an association of tramadol use with hypoglycemia when compared to patients taking codeine^18^. In a later case-control study this association was confirmed by Golightly *et al*. where patients taking tramadol were compared to patients on oxycodone^19^. Studies based on animal models have demonstrated that tramadol directly induced glucose utilization by hepatocytes and skeletal muscles of streptozotocin-induced diabetic rats via μ-opioid receptor activation^20,21^. Other animal studies have demonstrated the role of serotonin in glucose metabolism via insulin modulation^22,23^. Based on previous evidence from animal studies, tramadol induced hypoglycemia has been attributed to MOR agonism or serotonin modulation. Another possible etiology of hypoglycemia could be related to NMDAR antagonism^10,24–30^.

In this study we posed two questions: (1) is tramadol use significantly associated with an elevation of hypoglycemia reports in non-diabetic patients, (2) is hypoglycemia associated with any other opioids, SNRIs, or NMDAR modulators. SNRI and NMDAR modulators were selected as comparison patient treatment categories because they represent two non-opioid activities of tramadol.

Here we analyzed over twelve million ADR reports from United States FDA Adverse Event Reporting System (FAERS) and found a significant association of tramadol use with hypoglycemia. Among eleven opioids, four SNRIs and five NMDAR-antagonists that were analyzed, only methadone was associated with hypoglycemia similarly to tramadol.

## Methods

### FDA adverse event reporting system (FAERS/AERS)

Over twelve million adverse event reports were acquired from the FDA Adverse Event Reporting System (FAERS) and its older version Adverse Event Reporting System (AERS) data sets. At the time of the analysis the FAERS data set contained adverse effect reports from September 2012 to March 2019 and the AERS set contained data from January 2004 to August 2012. FAERS/AERS is a repository of post-marketing surveillance records on therapeutic agents reported to the FDA through MedWatch. The database consists of voluntary reports by pharmacists, physicians, patients, legal representatives, and other healthcare providers. Adverse events submitted directly to the manufacturer are legally required to be forwarded to FAERS/AERS.

Both FAERS and AERS data sets are available online at: http://www.fda.gov/Drugs/GuidanceComplianceRegulatoryInformation/Surveillance/AdverseDrugEffects/ucm082193.htm.

### Combining and normalizing the data

FAERS/AERS online reports were posted quarterly and were downloaded in sets of seven tables for each quarter in dollar separated text (.TXT) format. The data from the tables were extracted and joined into a consistent format for analysis. Demographic parameters were converted into single standard units to facilitate filtering and selections. The column names were unified and missing columns in older data sets were added with no values. The final version of the data set contained reports from the first quarter of 2004 to the first quarter of 2019. All international and domestic drug names of interest were translated to their corresponding United States Adopted Names Council approved generic names^31–33^.

### Cohort selection

A total of 12,004,552 FAERS/AERS reports were collected. Reports containing tramadol, codeine, hydrocodone, oxycodone, oxymorphone, hydromorphone, morphine, fentanyl, methadone, dextropropoxyphene, and tapentadol used as *monotherapy* were separated into their respective cohorts. Similarly, selection was performed for the following SNRIs: duloxetine, venlafaxine, desvenlafaxine, and milnacipran used as monotherapy, and drugs with NMDAR activity: minocycline, atomoxetine, ketamine, dextromethorphan, and memantine.

**Figure 1:**
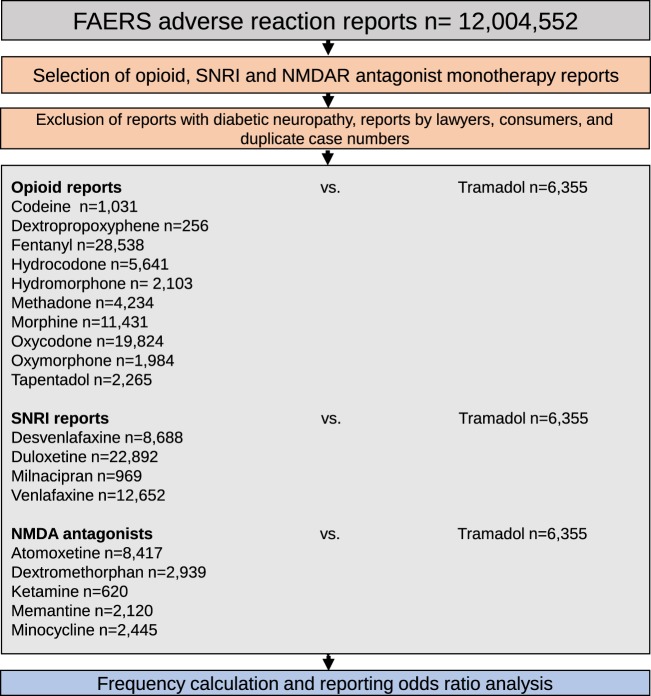
Inclusion, exclusion and analysis cohort selection for adverse event rate comparison between tramadol, non-tramadol opioid, SNRI and NMDAR antagonist cohorts.

Monotherapy was defined in these cases as reports where each patient was using only the medication of interest. A total of 145,404 monotherapy reports were analyzed: opioids (n = 83,662), SNRIs (n = 45,201), and NMDAR antagonists (n = 16,541). Reports where the diabetes indication was listed or where the medications were used to treat diabetic neuropathy were excluded (Fig. 1). Reports submitted by lawyers or consumers were excluded from the analysis due to higher potential for bias and misclassification. FAERS data sets included follow up reports with the same case identifier. These constituted 0.04% of the total reports and were also excluded from the analysis (Fig. 1). Demographic analysis was performed for tramadol, other opioid, SNRI, and NMDAR antagonist cohorts to illustrate the availability and the comparability of the chosen cohorts (Table 1).

**Table 1:** Patient demographics in tramadol, non-tramadol opioid, SNRI and NMDAR antagonist cohorts.

	Tramadol (n = 6,355)	Frequency (%)	Opioids (n = 77,307)	Frequency (%)	SNRIs (n = 45,201)	Frequency (%)	NMDAR antagonists (n = 16,541)	Frequency (%)
Female	3,035	47.8	36,439	47.1	30,663	67.8	8,081	48.9
Male	2,190	34.5	29,960	38.8	10,526	23.3	6,455	39.0
Unreported	1,130	17.8	10,908	14.1	4,012	8.9	2,005	12.1
Mean age, years (SD)	46.1 (22.7)		49.2 (21.3)		49.1 (18.4)		30.0 (24.7)	
Median age, years	51		52.7		49.8		47.3	
Unreported (%)	46.7		58.1		52.3		34.9	

Opioids included in this study were codeine (n = 1,031), dextropropoxyphene (n = 256), fentanyl (n = 28,538), hydrocodone (n = 5,641), hydromorphone (n = 2,103), methadone (n = 4,234), morphine (n = 11,431), oxycodone (n = 19,824), oxymorphone (n = 1,984), tapentadol (n = 2,265), and tramadol (n = 6,355).

SNRIs included in this study were desvenlafaxine (n = 8,688), duloxetine (n = 22,892), milnacipran (n = 969), venlafaxine (n = 12,652).

Drugs with NMDAR antagonist activity were atomoxetine (n = 8,417), dextromethorphan (n = 2,939), ketamine (n = 620), memantine (n = 2,120), and minocycline (n = 2,445).

Odd ratios were calculated using relative frequencies of hypoglycemia reports for tramadol when compared to other opioids, SNRIs and NMDAR antagonists. The term hypoglycemia was used because of its strict clinical definition (plasma glucose concentration below 70 mg/dL) and because it is the preferred MedDRA term used in FAERS reports. The common symptoms of hypoglycemia were not used for the search due to their variability, lower specificity, and wide presence in other disease states. The term ‘decreased blood glucose’ was not included in the search since it was much less frequent, and not equivalent to hypoglycemia since it may correspond to levels over 70 mg/dL. The query was performed with only one term ‘hypoglycemia’ in the ADR field for the selected monotherapy cohorts.

### Statistical analysis

#### Descriptive statistics

Frequencies for hypoglycemia ADRs were calculated by the equation:

1 $${\rm{Frequency}}=({\rm{Number}}\,{\rm{of}}\,{\rm{Records}}\,{\rm{with}}\,{\rm{ADR}})/({\rm{Number}}\,{\rm{of}}\,{\rm{Patient}}\,{\rm{Records}})\ast {\rm{100}}$$

#### Comparative statistics

ADR report rates were compared via the Reporting Odds Ratio (ROR) disproportionality analysis using the following equations:

2 $${\rm{ROR}}=({\rm{a}}/{\rm{b}})/({\rm{c}}/{\rm{d}})$$

where

a: Number of cases in exposed group with an adverse event.

b: Number of cases in exposed group with no adverse event.

c: Number of cases in control group with the adverse event.

d: Number of cases in control group with no adverse event.

3 $${\rm{LnROR}}=\,\mathrm{Ln}({\rm{ROR}})$$

Standard Error of Log Reporting Odds Ratio;

4 $${{\rm{SE}}}_{{\rm{LnROR}}}=\surd ({\rm{1}}/{\rm{a}}+{\rm{1}}/{\rm{b}}+{\rm{1}}/{\rm{c}}+{\rm{1}}/{\rm{d}})$$

95% Confidence Interval;

5 $$95 \% {\rm{CI}}=[\exp ({\rm{LnROR}}-1.96\times {{\rm{SE}}}_{{\rm{LnROR}}}),\,\exp ({\rm{LnROR}}+1.96\times {{\rm{SE}}}_{{\rm{LnROR}}})]$$

## Results

### Tramadol and hypoglycemia

**Figure 2:**
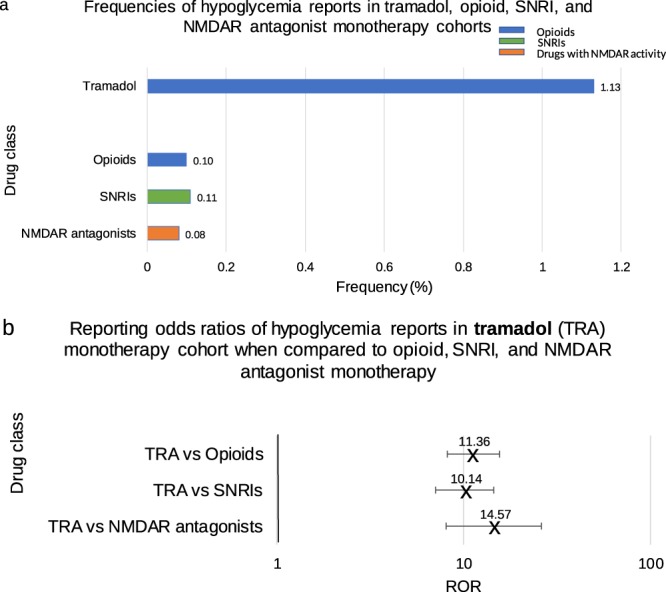
(**a**) Frequencies of hypoglycemia events for patients on tramadol (n = 6,355), opioids (n = 83,662), SNRIs (n = 45,201), and NMDAR antagonists (n = 16,541). (**b**) Odds ratios were calculated comparing frequencies of hypoglycemia reports from the tramadol cohort and each of the opioid, SNRI and NMDAR antagonist cohorts. Ranges represent 95% confidence intervals (95% CI) (see Methods). X-axis is presented in log scale. Abbreviations: TRA-tramadol, SNRI-serotonin norepinephrine reuptake inhibitor, NMDAR-N-methyl-D-aspartate receptor.

Frequencies of hypoglycemia reports were initially calculated for opioids, SNRIs and NMDAR antagonists as a class, for comparison with hypoglycemia reports in the tramadol cohort (Fig. 2a). There was a significant elevation in hypoglycemia reports in the tramadol cohort when compared to opioids-class: ROR 11.36, 95% confidence interval (CI) (8.23, 15.66), SNRIs-class: 10.14 (7.08, 14.54), and NMDAR antagonists-class 14.57 (8.07, 26.31) (Fig. 2b). This comparison emphasizes the special role of tramadol in causing hypoglycemia ADR unrelated to the pharmacology common to each of the studied drug classes.

This finding led us to study the individual drugs in each class which are known to have multitarget drug specific pharmacology and ADR profiles.

### Hypoglycemia in eleven individual opioid cohorts

**Figure 3:**
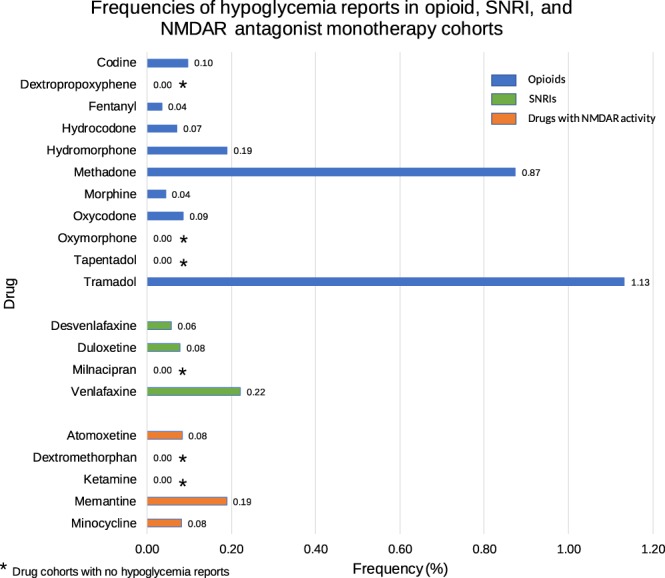
Frequencies of hypoglycemia events for patients on codeine (n = 1,030), dextropropoxyphene (n = 256), fentanyl (n = 28,538), hydrocodone (n = 5,641), hydromorphone (n = 2,103), methadone (n = 4,234), morphine (n = 11,431), oxycodone (n = 19,824), oxymorphone (n = 1,984), tapentadol (n = 2,265), tramadol (n = 6,355), desvenlafaxine (n = 8,688), duloxetine (n = 22,892), milnacipran (n = 969), venlafaxine (n = 12,652), atomoxetine (n = 8,417), dextromethorphan (n = 2,939), ketamine (n = 620), memantine (n = 2,120), and minocycline (n = 2,445).

**Figure 4:**
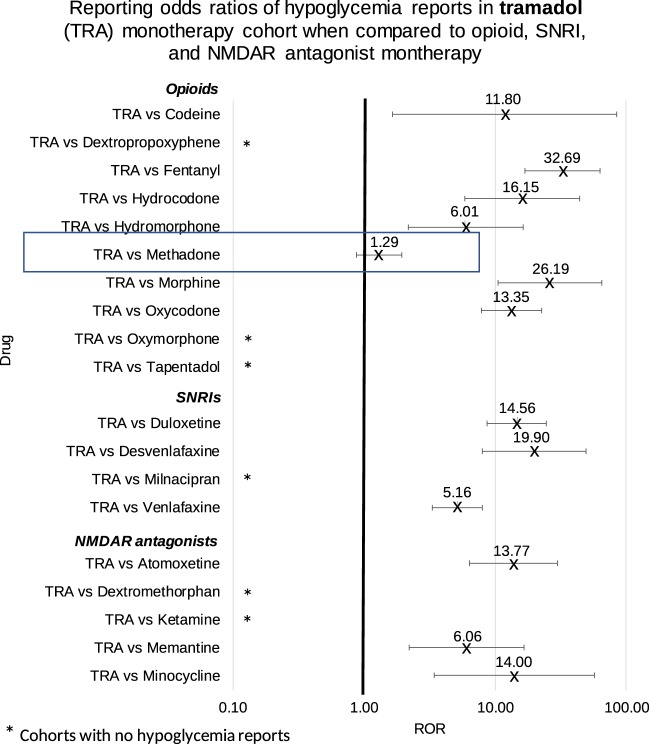
Reporting odds ratios were calculated comparing frequencies of hypoglycemia reports from the tramadol cohort and each of the opioid, SNRI and NMDAR antagonist cohorts. Ranges represent 95% confidence intervals (95% CI) (see Methods). X-axis is presented in log scale. Abbreviations: TRA-tramadol, SNRI-serotonin norepinephrine reuptake inhibitor, NMDAR-N-methyl-D-aspartate receptor.

Frequencies of FAERS/AERS hypoglycemia reports were calculated for each of the opioids (Fig. 3). Patients who used tramadol as monotherapy had a significant elevation in the frequency of hypoglycemia when compared to nine opioids with mean ROR values ranging from 6 to 33 (Table 2 and Fig. 4). Interestingly, no significant difference was found in hypoglycemia frequencies between tramadol and methadone cohorts with 95% CI covering the value of 1: ROR. Not a single report with the hypoglycemia ADR was found in the tapentadol, oxymorphone, and dextropropoxyphene cohorts.

**Table 2:** Reporting odds ratios were calculated comparing frequencies of hypoglycemia reports from the tramadol cohort and each of the individual drugs in the opioid, SNRI and NMDAR antagonist cohorts.

Drug	ROR	95% CI
**Opioids**		
TRA vs Codeine	11.80	[1.64, 85.03]
TRA vs Dextropropoxyphene	*	
TRA vs Fentanyl	32.69	[16.86, 63.38]
TRA vs Hydrocodone	16.15	[5.89, 44.23]
TRA vs Hydromorphone	6.01	[2.19, 16.48]
TRA vs Methadone	1.29	[0.87, 1.93]
TRA vs Morphine	26.19	[10.57, 64.86]
TRA vs Oxycodone	13.35	[7.86, 22.67]
TRA vs Oxymorphone	*	
TRA vs Tapentadol	*	
**SNRIs**		
TRA vs Desvenlafaxine	19.90	[8.03, 49.29]
TRA vs Duloxetine	14.56	[8.68, 24.43]
TRA vs Milnacipran	*	
TRA vs Venlafaxine	5.16	[3.34, 8.00]
**NMDAR antagonists**		
TRA vs Atomoxetine	13.77	[6.33, 29.93]
TRA vs Dextromethorphan	*	
TRA vs Ketamine	*	
TRA vs Memantine	6.06	[2.22, 16.61]
TRA vs Minocycline	13.99	[3.43, 57.10]

Ranges represent 95% confidence intervals (95% CI) (see Methods). *Represents cohorts with no hypoglycemia reports.

### Hypoglycemia in four individual SNRI cohorts

Each of the SNRIs were analyzed for hypoglycemia report frequencies.

Patients who used tramadol as monotherapy had a significant elevation in the frequency of hypoglycemia when compared to patients taking each of the four SRNIs with mean ROR values ranging from 5 to 20. The milnacipran cohort did not have any reports of hypoglycemia. (Table 2 and Fig. 4).

### Hypoglycemia in five NMDAR antagonist reports

Reports where tramadol was used had a significant elevation in the frequency of hypoglycemia when compared to patients taking each of the five drugs with NMDAR antagonist activity with mean ROR values in the range of 6–14. The ketamine and dextromethorphan cohorts did not have any reports of hypoglycemia ADR (Table 2 and Fig. 4).

### Co-occurring ADRs

The top ADRs co-occurring with hypoglycemia were relatively rare but consistent hypoglycemia for tramadol. These included ‘loss of consciousness’ and ‘hypoglycemic coma’ (Table 3).

**Table 3:** ADRs co-occurring with hypoglycemia in the tramadol monotherapy cohort.

ADRs co-occurring with hypoglycemia	%
Hypoglycemia	100.00
Convulsion	22.89
Toxicity to various agents	16.87
Loss of consciousness	12.05
Overdose	10.84
Depressed level of consciousness	10.84
Vomiting	7.23
Malaise	7.23
Intentional overdose	7.23
Suicide attempt	6.02
Suicidal ideation	6.02
Seizure	6.02
Hypoglycemic coma	6.02
Hypoxia	4.82
Road traffic accident	3.61
Hypotension	3.61
Hyperhydrosis	3.61
Neonatal drug withdrawal syndrome	3.61
Dizziness	3.61
Altered state of consciousness	3.61
Accidental overdose	3.61

ADR occurrences over 3% are reported.

### Comparing methadone with ten other opioids and NMDAR antagonists

**Figure 5:**
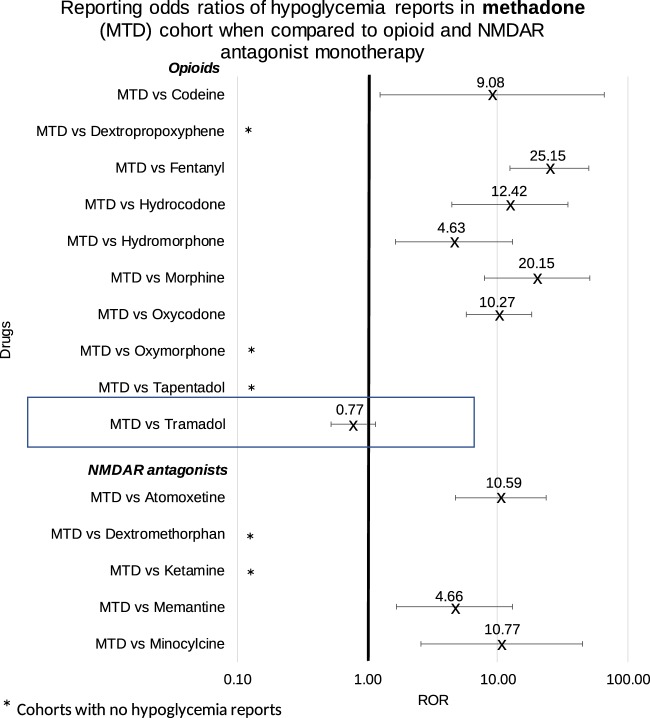
Reporting Odds ratios were calculated comparing frequencies of hypoglycemia reports from the methadone cohort and each of the opioid and NMDAR antagonist cohorts. Ranges represent 95% confidence intervals (95% CI) (see Methods). X-axis is presented in log scale. Abbreviations: MTD-methadone, NMDAR-N-methyl-D-aspartate receptor.

Similar analysis was performed to evaluate hypoglycemia report frequency in the methadone monotherapy cohort (Fig. 5). Methadone’s analgesic effect is attributed to MOR agonism^34,35^ and NMDAR antagonism^27,36^. Patients who used methadone as monotherapy had a significant elevation in the frequency of hypoglycemia when compared to nine (non-tramadol) opioids (mean ROR in the range of 4 to 26), and five other drugs with NMDAR antagonist activity (mean ROR in the range of 4 to 11) (Table 4 and Fig. 5). As expected there was no significant difference between hypoglycemia reports in the methadone cohort when compared to the tramadol cohort. Similarity in the ROR profile between both tramadol and methadone vs other drugs in the same class further supports a mechanism of hypoglycemia unrelated to their common class-wide mechanisms of action.

**Table 4:** Reporting Odds ratios were calculated comparing frequencies of hypoglycemia reports from the methadone cohort and each of the opioid and NMDAR antagonist cohorts.

Drug	ROR	95% CI
**Opioids**		
MTD vs Codeine	9.08	[1.24, 66.25]
MTD vs Dextropropoxyphene	*	
MTD vs Fentanyl	25.15	[12.50, 50.61]
MTD vs Hydrocodone	12.42	[4.43, 34.88]
MTD vs Hydromorphone	4.63	[1.65, 13.00]
MTD vs Morphine	20.15	[7.91, 51.29]
MTD vs Oxycodone	10.27	[5.78, 18.26]
MTD vs Oxymorphone	*	
MTD vs Tapentadol	*	
MTD vs Tramadol	0.77	[0.52, 1.15]
**NMDAR antagonists**		
MTD vs Atomoxetine	10.59	[4.72, 23.78]
MTD vs Dextromethorphan	*	
MTD vs Ketamine	*	
MTD vs Memantine	4.66	[1.66, 13.10]
MTD vs Minocycline	10.77	[2.59, 44.72]

Ranges represent 95% confidence intervals (95% CI) (see Methods). *Represents cohorts with no hypoglycemia reports.

### Co-occurring ADRs

Interestingly, the co-occurring (non-hypoglycemia-related) ADRs for methadone (Table 5), were mostly of cardiovascular nature. Hypoglycemia related ADRs were ‘hyper-insulinemic hypoglycemia’ and ‘increased blood insulin’. The overlapping ADRs were consistent with opioid toxicity.

**Table 5:** ADRs co-occurring with hypoglycemia in the methadone monotherapy cohort.

ADRs co-occurring with hypoglycemia	%
Hypoglycemia	100.00
Hypotension	31.70
Respiratory failure	26.83
Miosis	21.95
Accidental overdose	14.63
QT prolongation	12.20
Depressed level of consciousness	12.20
Coma	12.20
Sinus tachycardia	9.76
Respiratory depression	9.76
Pneumonia	9.76
Involuntary muscle contractions	9.76
Hyperinsulinemic hypoglycemia	9.76
Cyanosis	9.76
Accidental exposure to product by child	9.76
Accidental exposure to product	9.76
Ventricular extrasystoles	7.32
Unresponsive to stimuli	7.32
Somnolence	7.32
Intentional overdose	7.32
Hypoventilation	7.32
Blood insulin increased	7.32
Abnormal respiration	4.88
Overdose	4.88
Muscle tightness	4.88
Mental disorder	4.88
Bradypnea	4.88
Blood glucose decreased	4.88
Adrenal insufficiency	4.88

Frequencies over 3% reported.

## Discussion

To our knowledge, this study was the first analysis of the FDA Adverse Event Reporting System (FAERS) and its older version Adverse Event Reporting System (AERS) to generate a risk profile of tramadol’s association with hypoglycemia when compared to other opioids, SNRIs, and NMDAR modulators. In this study we quantified the association between tramadol exposure and hypoglycemia. By utilizing a total of 145,404 monotherapy reports for twenty therapeutics, we compared the reporting odds ratios of hypoglycemia reports and identified two drugs, tramadol and methadone, with higher risk. We were able to confirm the previous association studies of tramadol vs hypoglycemia and the lack of that association with oxycodone and codeine^18,19^. Additionally, we provided the evidence for no significant elevation of hypoglycemia ADRs in nine other opioids with the single significant exception of methadone. The hypothesis of SNRI or NMDAR relation to hypoglycemia led us to analyze the related drugs. To our surprise we found no evidence of significant elevation in hypoglycemia reports in the SNRI and NMDAR antagonist cohorts. These findings imply that opioid receptor agonism, serotonin and norepinephrine reuptake, and N-methyl-D-aspartate receptor antagonism alone did not correlate with elevation in hypoglycemia reports suggesting a subtler mechanism specific to tramadol and methadone.

Methadone use was associated with hypoglycemia in a study using animal models, where methadone significantly decreased blood glucose levels in a dose-dependent manner, while morphine, fentanyl, levorphanol, oxycodone or morphine-6β-glucuronide did not show significant change from baseline glucose levels^37^. Furthermore some case reports^38,39^, and retrospective studies^40^ also show evidence of hypoglycemia association with methadone use.

Most of the ADRs co-occurring with hypoglycemia reports in the tramadol and methadone cohorts, shown in Tables 3 and 5, were common to the opioid class (depressed level of consciousness, vomiting, malaise, dizziness, respiratory failure, miosis etc.) or hypoglycemia related (decreased blood glucose, hypoglycemic coma), except for side effects unique to tramadol (convulsions, seizure), or methadone (QT prolongation, sinus tachycardia). Furthermore, methadone co-occurring ADRs included ‘hyper-insulinemic hypoglycemia’ and ‘increased blood insulin’, which may indicate one of the mechanisms of the observed hypoglycemia ADR. The full etiology of hypoglycemia for both tramadol and methadone needs further studies.

## Conclusion

In our study we observed increased risk of hypoglycemia ADRs in FAERS reports of tramadol with respect to other opioid, SNRI, and NMDAR modulating drug reports in patients without concurrent medication use and comorbidities. We observed a similar association between methadone monotherapy and hypoglycemia. It may be beneficial to monitor glucose levels when initiating tramadol or methadone in both diabetic and non-diabetic patients. Alternative opioids or non-opioid pain medications may be safer to use with patients at risk of hypoglycemia or any complications associated with hypoglycemia.

### Study limitations

FDA FAERS/AERS reporting is voluntary. The calculated frequencies do not represent actual population frequencies. A recent study found that FAERS/AERS reporting can be biased by legal or scientific variables as well as newsworthiness^41^. Another study has shown that FAERS/AERS reporting can be significantly underreported for some drugs^42^. Absence of comprehensive medical records and lab values further limits the scope of our analysis. Some concurrent medications and comorbidities may be missing from the records due to underreporting which may introduce uncertainties in ADR frequencies, and reporting odds ratios. We cannot derive the physiological mechanism of the adverse event from the FAERS/AERS records. The reporting odds ratios represent frequency ratios of reported adverse effects and are not based on population incidences. As with any association study, causality cannot be inferred from association. The reported cases were not clinically evaluated for causality by experts.
